# Supplementary material for: Episiotomy practices and associated factors in central Vietnam
Source: PLoS One. 2026 Feb 25;21(2):e0321002. doi: 10.1371/journal.pone.0321002 (PMC12935254; doi:10.1371/journal.pone.0321002)
Supplement: Attachment — Submitted filename: Response_Letter-v1.docx [file pone.0321002.s002.docx]

**Dr. Chenchit Pichailuck, Associate Professor**

**Academic Editor**

**PLOS ONE**

*Re:* *Episiotomy Practices and Associated Factors in Central Vietnam*

Date: April 18, 2025,

Dear Dr. Pichailuck,

Thank you very much for the opportunity to revise this manuscript. We have addressed the comments in the point-by-point response below.

Both tracked-changes version and a clean version of the revised manuscript are included.

We sincerely appreciate your valuable feedback and suggestions, which have significantly improved the quality of our work.

We hope that our revision has met PLOS ONE’s publication criteria and look forward to your response!

Yours sincerely,

Drs. Hoang Thi Nam Giang & Thanh-Huyen T. Vu

**Editor Comments**

***Comment****: The rationale of the study is not clearly stated. The national policy of Veitnam to reduce episiotomy practice has been launched one year prior to the data collection. The readers may be curious to know how things have been done. Moreover, why the auhtors choose 'one year following the policy' to represent the situation when there could some consequential effects from prior-to-policy practice.*

**Response**: We have revised the introduction to clarify the rationale behind our study. Currently, there is limited data on episiotomy practices in Vietnam and no data on the factors associated with this procedure. While a few studies have reported episiotomy prevalence in Northern and Southern Vietnam, none have been done in Central Vietnam; and none have focused on identifying the contributing factors. Our study aims to help fill this gap and we hope that our results can provide a foundation for future research on the complications and outcomes of episiotomy among Vietnamese mothers.

The study is based on previously collected data, and unfortunately, we do not have data for subsequent years. We discussed this issue as one of our limitations. We also removed the mention of the national policy to avoid misleading readers about its impact, as this could not be adequately addressed in this current study. The revision as follows:

**Introduction: Page 4 (line 65-67) and page 5 (line 68-82):**

“In Vietnam, the rate of episiotomy use and its associated factors are rarely reported despite the 2014 policy change that no longer recommends routine episiotomy(8). There was a survey conducted between 2012-13 in a maternity hospital in southern Vietnam to assess clinicians’ practices regarding episiotomy (9). In this study, among nulliparous women, the majority of obstetricians (82.6%) and midwives (98.7%) reported using episiotomies more than 90% of the time. Among multiparous women, a quarter of obstetricians reported using episiotomies under 60% of the time, while this rate for midwives was 3.8%. In 2020, the rate of episiotomy was reported in a study conducting that focusing on breastfeeding in both southern and northern Vietnam, where 83.3% of mothers self-reported having undergone a vaginal birth with an episiotomy (10).

To our knowledge, no comprehensive study has been conducted on episiotomy practices and their associated factors in Vietnam, particularly in Central Vietnam. Therefore, this study aimed to describe the prevalence of episiotomy among women who had vaginal births of single, full-term infants, and to identify the maternal and infant factors associated with this practice.”

**Page 15 (line 235-236)**

“The data used in this study were collected in 2015-2016 and therefore may not reflect current episiotomy practices”.

***Comment****: The conclusion of the abstract shows that 'one year after the implementation of..' comes without background information.*

**Response**: We have removed the reference to policy information and added further details on modifiable factors associated with episiotomy practice in the conclusion, as follows:

**Page 3 (line 34-38)**

“This study highlights a very high episiotomy rate in Central Vietnam, especially among primiparous women. Several modifiable maternal and neonatal factors are linked to episiotomy use in multiparous women. Comprehensive research and targeted interventions are needed to reduce episiotomy rates, particularly among first-time mothers in Vietnam.”

***Comment****: Page 4 Line 51: I doubt with the advantages of episiotomy over spontaneous tear in that 'healing is better', 'preserve relaxation of the pelvic floor m.', I think that it should be the opposite direction.*

**Response**: We have removed these references.
