# Supplementary material for: Episiotomy practices and associated factors in central Vietnam
Source: PLoS One. 2026 Feb 25;21(2):e0321002. doi: 10.1371/journal.pone.0321002 (PMC12935254; doi:10.1371/journal.pone.0321002)
Supplement: Attachment — Submitted filename: Response_Letter-v2.docx [file pone.0321002.s003.docx]

**Dr. Chenchit Pichailuck, Associate Professor**

**Academic Editor**

**PLOS ONE**

*Re:* *Episiotomy Practices and Associated Factors in Central Vietnam*

Date: November 11, 2025,

Dear Dr. Pichailuck,

Thank you very much for the opportunity to revise this manuscript. We have addressed the comments in the point-by-point response below.

Both tracked-changes version and a clean version of the revised manuscript are included.

We sincerely appreciate your valuable feedback and suggestions, which have significantly improved the quality of our work.

We hope that our revision has met PLOS ONE’s publication criteria and look forward to your response!

Yours sincerely,

Drs. Hoang Thi Nam Giang & Thanh-Huyen T. Vu

**Editor Comments**

***Comment****: Episiotomy has been commonly practiced in both Thailand and Veitnam because the procedure is included in the Medical curriculum whereas the global trend goes to the opposite direction. Many countries in this region such as Malaysia and Singapore minimize the procedure by increasing the perineal preparation such as massaging, breathing exercise during antenatal care, which takes extra time and effort. The high prevalence of episiotomy in Veitnam can be lessened by the strong Government policy which was mentioned in the original version of manuscript. And, I think it would be very interesting to learn from how the Veitnamese Government deal with the problem. However, the authors decide to remove this part resulting in lower added value of the study.*

**Response**: We are sorry for misunderstanding your comments. We have now added information on the government policy in the abstract, introduction and the discussion.

The revision as follows:

**Abstract: Page 3 (line 35-42)**

“This study highlights a very high episiotomy rate in Central Vietnam, especially among primiparous women despite recommendations from the World Health Organization and the Viet Nam Ministry of Health. Several modifiable maternal and neonatal factors are linked to episiotomy use in multiparous women. While one year may not be sufficient to fully assess the restrictive policy's effect, the results of our study also suggest that the policy has not had a significant impact on reducing the episiotomy rate after one year. This indicates that, in addition to the policy, further support and interventions are needed to reduce episiotomy rate”

**Introduction: Page 4 (line 64-66) and page 5 (line 67-86)**

“In Vietnam, the rate of episiotomy use and its associated factors are rarely reported despite the 2014 policy change that no longer recommends routine episiotomy (8). Prior to 2014, only one survey conducted between 2012-13 in a maternity hospital in southern Vietnam to assess clinicians’ practices regarding episiotomy (9). In this study, among nulliparous women, the majority of obstetricians (82.6%) and midwives (98.7%) reported using episiotomies more than 90% of the time. Among multiparous women, a quarter of obstetricians reported using episiotomies under 60% of the time, while this rate for midwives was 3.8%. In 2014, the Vietnam Ministry of Health approved a technical guide on essential care for mothers and newborns during and immediately after birth (EENC), the practice of routine episiotomy is no longer recommended (8). Since then, there is lacking data to evaluate the effect of this restrictive recommendation. In 2020, the rate of episiotomy was reported in a study conducting that focusing on breastfeeding in both southern and northern Vietnam, where 83.3% of mothers self-reported having undergone a vaginal birth with an episiotomy (10).”

“To our knowledge, no comprehensive study has been conducted on episiotomy practices and their associated factors in Vietnam, particularly in Central Vietnam. Moreover, although routine episiotomy has not been recommended since the implementation of EENC guideline in 2014, and EENC was introduced in Da Nang Hospital for Women and Children the same year, data on episiotomy practice during the early stage of this transition remain limited. Therefore, this study aimed to describe the prevalence of episiotomy among women who had vaginal births of single, full-term infants after 1-year period of the implementation of restrictive episiotomy guidelines in Vietnam, and to identify the maternal and infant factors associated with this practice”.

**Discussion: Page 12 (line 176-177), page 12, 13 (line 182-185), page 15 (line 240-241), and page 16 (line 250-253)**

“This study included 3,471 singleton, full-term vaginal births in Central Vietnam during 2015-2016, one year following the implementation of restrictive episiotomy guidelines launched in the country”

“The high episiotomy rate in our study aligns with the finding from existing literature in other regions of the country. A survey conducted in 2012-2013 among obstetricians and midwives in southern Vietnam - prior to the implementation of restrictive episiotomy guidelines by the Vietnam Ministry of Health in 2014 - reported that 82.6% of obstetricians and 98.7% midwives performed episiotomies more than 90% of the time for first-time births (9)”

“Strengths of our study include access to delivery data from hospital birth registers at the largest maternity hospitals in Central Vietnam, where restrictive episiotomy guidelines were first introduced in 2014”

“The data used in this study were collected in 2015-2016, only one year following restrictive policy of episiotomy, which may not be a sufficient time to assess the effect of the policy. Also, our data may not reflect current episiotomy practices”

***Comment****: According to the study, episiotomy is commonly practicec in all deliveries. I am not sure if around 30% of multiparous women who did not undergo episiotomy had too short second stage to receive episiotomy or not. Second, third stage of labour; post-partum hemorrhage and instrumental deliveries should be taken into consideration.*

**Response**: The study is based on previously collected data, and unfortunately we do not have data on the duration of second and third stage of labour and other information to add in the results.

***Comment****: Conceptual framework of the study should be re-considered.*

**Response**: Thank you for your suggestion. We have added goverment policy as an important part in our manuscript.
