# Supplementary material for: Episiotomy practices and associated factors in central Vietnam
Source: PLoS One. 2026 Feb 25;21(2):e0321002. doi: 10.1371/journal.pone.0321002 (PMC12935254; doi:10.1371/journal.pone.0321002)
Supplement: Attachment — Submitted filename: Response_Letter-v4.docx [file pone.0321002.s004.docx]

**Dr. Berhanu Elfu Feleke, Ph.D**

**Academic Editor**

**PLOS One**

*Re:* *Episiotomy Practices and Associated Factors in Central Vietnam*

Date: 2 January 2026

Dear Dr. Berhanu Elfu Feleke,

Thank you very much for the opportunity to revise this manuscript. We have addressed the comments in the point-by-point response below.

Both tracked-changes version and a clean version of the revised manuscript are included.

We sincerely appreciate your valuable feedback and suggestions, which have significantly improved the quality of our work.

We hope that our revision has met PLOS One’s publication criteria and look forward to your response!

Yours sincerely,

Drs. Hoang Thi Nam Giang & Thanh-Huyen T. Vu

**Editor Comments**

***Comment****: Please report interval estimates (e.g., 95% confidence intervals) consistently for all key effect measures to allow readers to assess the precision of the estimates.*

**Response**: We reported 95% confidence intervals for all key effect measures.

The revisions are as follows:

**Abstract: Page 2 (line 23-27)**

“Among 3,471 eligible singleton births, 2,770 mothers (79.8%, 95% CI: 78.4% - 81.1%) underwent an episiotomy. The episiotomy rate was significantly higher in first-time births (97.7%, 95% CI: 96.8% - 98.3%) compared to second or subsequent births (61.5%, 95% CI: 59.2% - 63.8%), p<0.001”

**Result: Page 8 (line 145-147)**

“Among the 3,471 births, the mean maternal age was 28.4, 2,770 (79.8%, 95% CI: 78.4% - 81.1%) involved an episiotomy. The episiotomy rate was 97.7% for first-time births (95% CI: 96.8% - 98.3%) and 61.5% for second or subsequent births (95% CI: 59.2% - 63.8%)”

**Table 1: Page 9-11**

**Table 1**. Maternal and Neonatal Characteristics, Overall and by Parity, n=3,471

| **Characteristics** | **All**  (n=3,471) | **First-time births** (n=1,756) | **Second or subsequent births** (n=1,715) | **P-value*** |
| --- | --- | --- | --- | --- |
| **Maternal characteristics** | | | | |
| **Age** (year) | | | | |
| Mean (95% CI) | 28.4 (28.2 – 28.5) | 25.9 (25.7 – 26.0) | 30.9 (30.7 – 31.1) | <0.001 |
| **Occupation,** n (%, 95% CI) | | | | |
| High physical activity | 1490 (42.9, 41.1 – 44.6) | 735 (41.9, 39.5 – 44.2) | 755 (44.0, 41.7 – 46.4) | 0.031 |
| Moderate physical activity | 788 (22.7, 21.3 – 24.1) | 381 (21.7, 19.8 – 23.7) | 407 (23.7, 21.7 – 25.8) |  |
| Low physical activity | 1193 (34.4, 32.8 – 36.0) | 640 (36.4, 34.2 – 38.7) | 553 (32.3, 30.0 – 34.5) |  |
| **History of miscarriage,** n (%, 95% CI) | | | | |
| No | 2994 (86.3, 85.1 – 87.4) | 1601 (91.2, 89.7 – 92.5) | 1393 (81.2, 79.3 – 83.0) | <0.001 |
| Yes | 477 (13.7, 12.6 – 14.9) | 155 (8.8, 7.5 – 10.3) | 322 (18.8, 17.0 – 20.7) |  |
| **Neonatal characteristics** | | | | |
| **Neonatal birth weight** (grams) | | | | |
| Mean (95% CI) | 3128.9  (3116.8 – 3141.0) | 3052.5  (3036.8 – 3068.2) | 3207.1  (3189.3 – 3224.9) | <0.001 |
| **Catergory**, n (%, 95% CI) |  |  |  |  |
| Quartile 1: <2900 | 767 (22.1, 20.7 – 23.5) | 484 (27.6, 25.5 – 29.7) | 283 (16.5, 14.8 – 18.3) | <0.001 |
| Quartile 2: 2900 to <3100 | 701 (20.2, 18.9 – 21.6) | 418 (23.8, 21.8 – 25.9) | 283 (16.5, 14.8 – 18.3) |  |
| Quartile 3:3100 to <3400 | 1100 (31.7, 30.1 – 33.3) | 540 (30.7, 28.6 – 33.0) | 560 (32.7, 30.4 – 34.9) |  |
| Quartile 4: ≥3400 | 903 (26.0, 24.6 – 27.5) | 314 (17.9, 16.1 – 19.8) | 589 (34.3, 32.1 – 36.6) |  |
| **Neonatal sex**, n (%, 95% CI) | | | | |
| Male | 1755 (50.6, 48.9 – 52.2) | 882 (50.2, 47.9 – 52.6) | 873 (50.9, 48.5 – 53.3) | 0.690 |
| Female | 1716 (49.4, 47.8 – 51.1) | 874 (49.8, 47.4 – 52.1) | 842 (49.1, 46.7 – 51.5) |  |
| **Episiotomy rate**, n (%, 95% CI) | | | | |
| No | 701 (20.2, 18.9 – 21.6) | 41 (2.3, 1.7 – 3.2) | 660 (38.5, 36.2 – 40.8) | <0.001 |
| Yes | 2770 (79.8, 78.4 – 81.1) | 1715 (97.7, 96.8 – 98.3) | 1055 (61.5, 59.2 – 63.8) |  |

*P-values for overall group comparisons based on T-tests or Chi-square tests; CI: confidence interval

***Comment****: Please clarify who performed the episiotomies (e.g., midwives versus obstetricians/gynaecologists). If these data are available, consider stratifying episiotomy rates by provider type, as this may provide important insights into clinical practice patterns.*

**Response**: We do not have data that allows stratification of episiotomy rates by provider type, and we acknowledge this is a limitation of our study. We have added further details to the Limitation section accordingly.

The revision is as follows:

**Discussion: Page 16 (line 248-255)**

“However, there are several limitations to this study. First, we lacked information on the duration of the second stage of labor, episiotomy-related complications, and perineal tear rates among women who did not undergo episiotomy. This limitation may restrict our ability to assess the full range of potential risk factors for episiotomy and limit the interpretation of episiotomy use and its relationship with maternal birth outcomes. Second, the absence of data allowing stratification of episiotomy rates by maternity care providers may limit insights into differences in clinical practice patterns”

***Comment****: If available, please report the perineal tear rates among women who did not undergo episiotomy (n = 701). This information would strengthen the interpretation of episiotomy use and its relationship to maternal birth outcomes.*

**Response**: As noted in the Limitation section (page 16, line 248-255) and in our previous response, we do not have data on perineal tear rates among women who did not undergo episiotomy in our study population. We agree that the absence of this information limits the interpretation of episiotomy use and its relationship with maternal birth outcomes. However, data from Hung Vuong hospital in southern Vietnam, based on a review of 367 post-partum medical records of women who received perineal protection, showed that 77.9% experienced perineal tear; among these, 94% were first-degree tears, and no third- or fourth-degree tears were reported. These finding suggest that perineal tears without episiotomy are predominantly mild and support the need for further comprehensive research to inform strategies aimed at reducing the high rate of episiotomy in Vietnam. We have included these comments in our discussions (page 16 lines 260-262 and page 17 lines 263-265).

“Together with findings from Hung Vuong hospital in southern Vietnam suggesting that perineal tears without episiotomy are predominantly mild if women received perineal protection (25), our findings lay the groundwork for future comprehensive research focusing on the complications and outcomes of episiotomy among Vietnamese mothers to inform strategies aimed at reducing the high rate of episiotomy in Vietnam”
